# Supplementary material for: Unraveling the differential dynamics of developmental fate in central and peripheral nervous systems
Source: Sci Rep. 2016 Nov 2;6:36397. doi: 10.1038/srep36397 (PMC5090986; doi:10.1038/srep36397)
Supplement: Supplementary Information [file srep36397-s1.pdf]

Supplementary Information

Unraveling the differential dynamics of developmental fate in central and peripheral nervous systems

Dola Sengupta[1], Sandip Kar[1,*]

Supplementary Figures

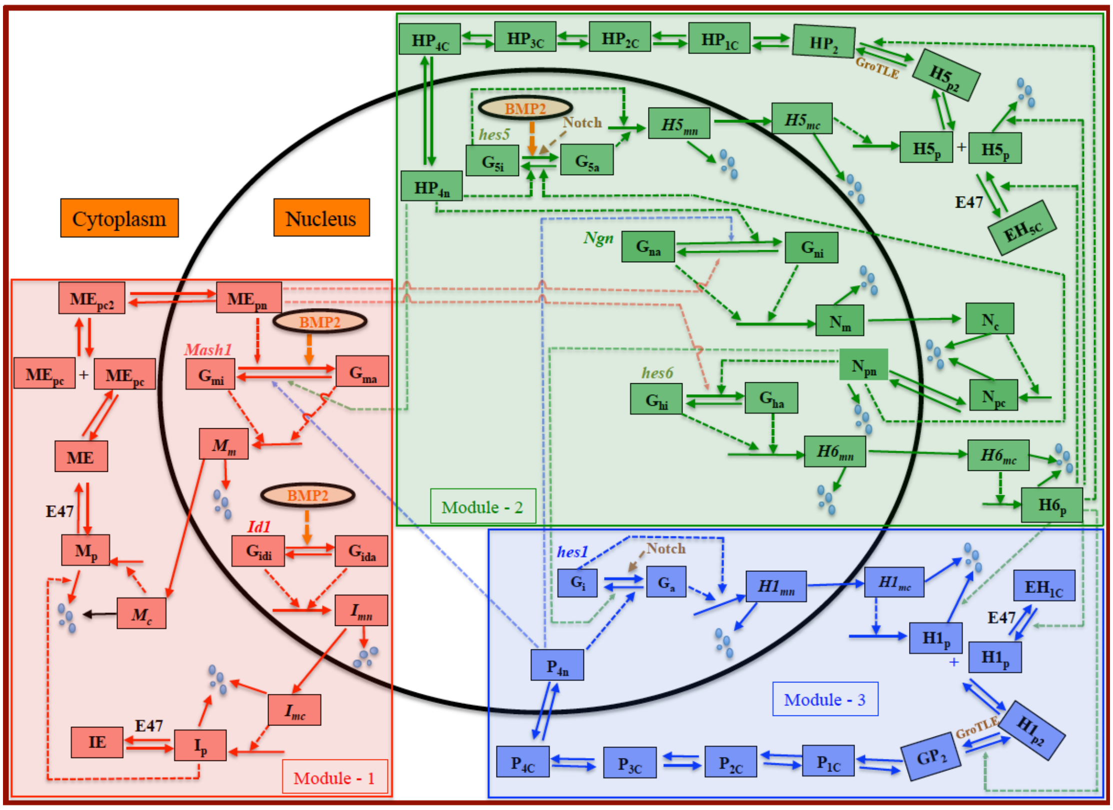

Supplementary Figure 1. Complete neuronal differentiation regulatory network of NSC's in CNS and PNS. (Arrows (Solid - intra modular, dashed – inter modular) and hammer-headed lines (Solid - intra modular, dashed – inter modular) represent direct or indirect activation and inhibition processes respectively.) The model consists of three modules (Red color region – module-1, Green color region – module-2 and Blue color region – module-3) and incorporates the effect of BMP2, which dynamically controls the developmental fates of NSC's in CNS and PNS. The detailed regulatory interactions are described in *SI Appendix* where comprehensive schemes for the whole interaction network and three modules are discussed elaborately. Corresponding kinetic equations, description of the variables and parameters are depicted in SI Text.

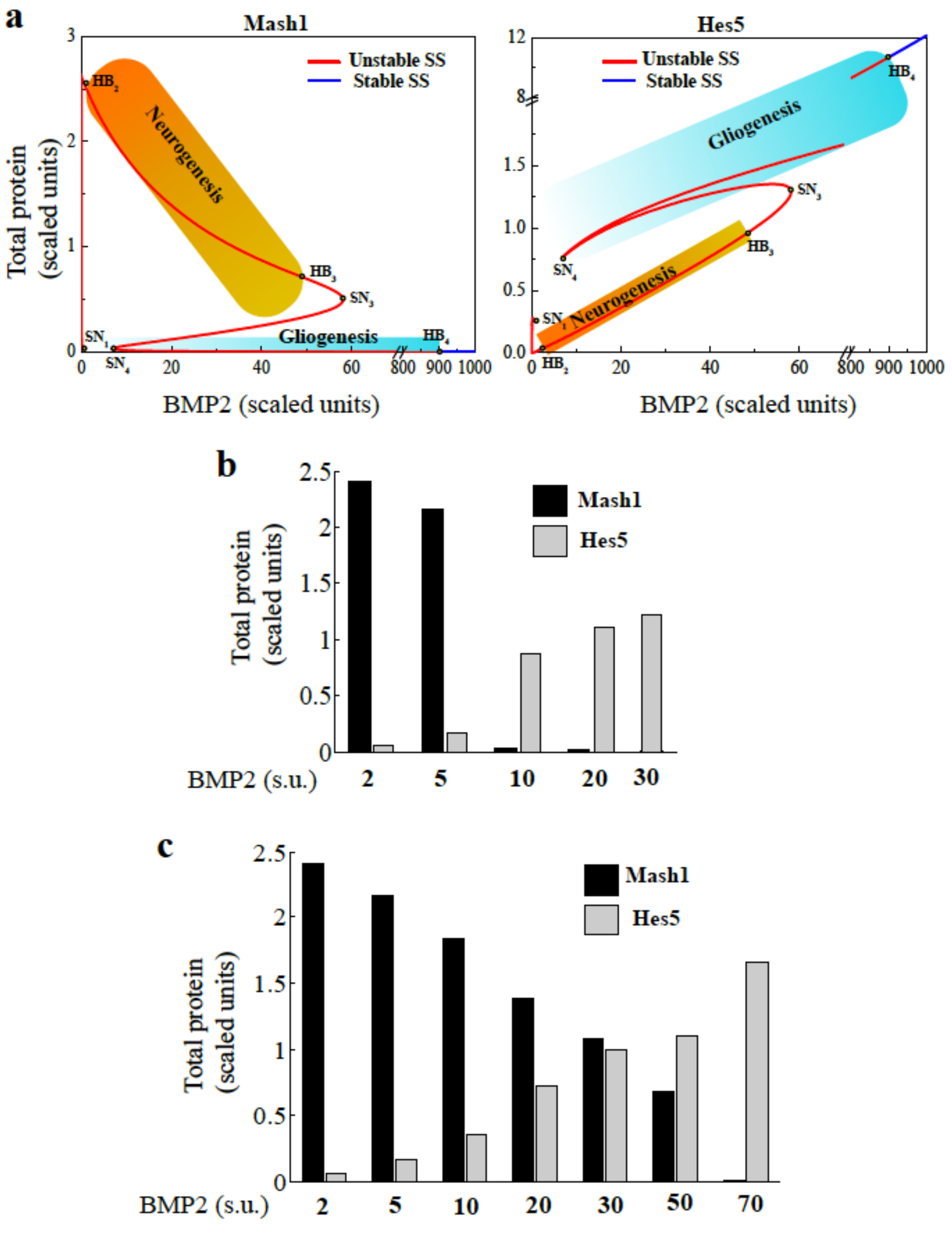

Supplementary Figure 2. Total Mash1 and Hes5 proteins as a function of BMP2 in CNS. (a) Bifurcation diagrams of total Mash1 and Hes5 proteins are plotted as a function of BMP2 (both the axes are shown in linear scale). Increasing the level of BMP2 drives the developmental cell fate from neurogenic state (high Mash1 and low Hes5 expressions, shown by the graded orange region) to gliogenic state (low Mash1 and high Hes5 expressions, shown by the graded blue region). The parameter values are given in SI Text. **(b)** Total Mash1 and Hes5 protein expressions with sudden change in BMP2 concentration from a very low level of BMP2. **(c)** Total Mash1 and Hes5 protein expressions with gradual increase in BMP2 concentration starting from BMP2=2 s.u.. After initializing the system at BMP2=2 s.u., BMP2 concentration is gradually increased upto 70 s.u.. Since all the

proteins are showing oscillatory behavior under the range of BMP2 doses employed, we plotted the expression levels of the individual proteins by taking the average of the oscillation amplitude.

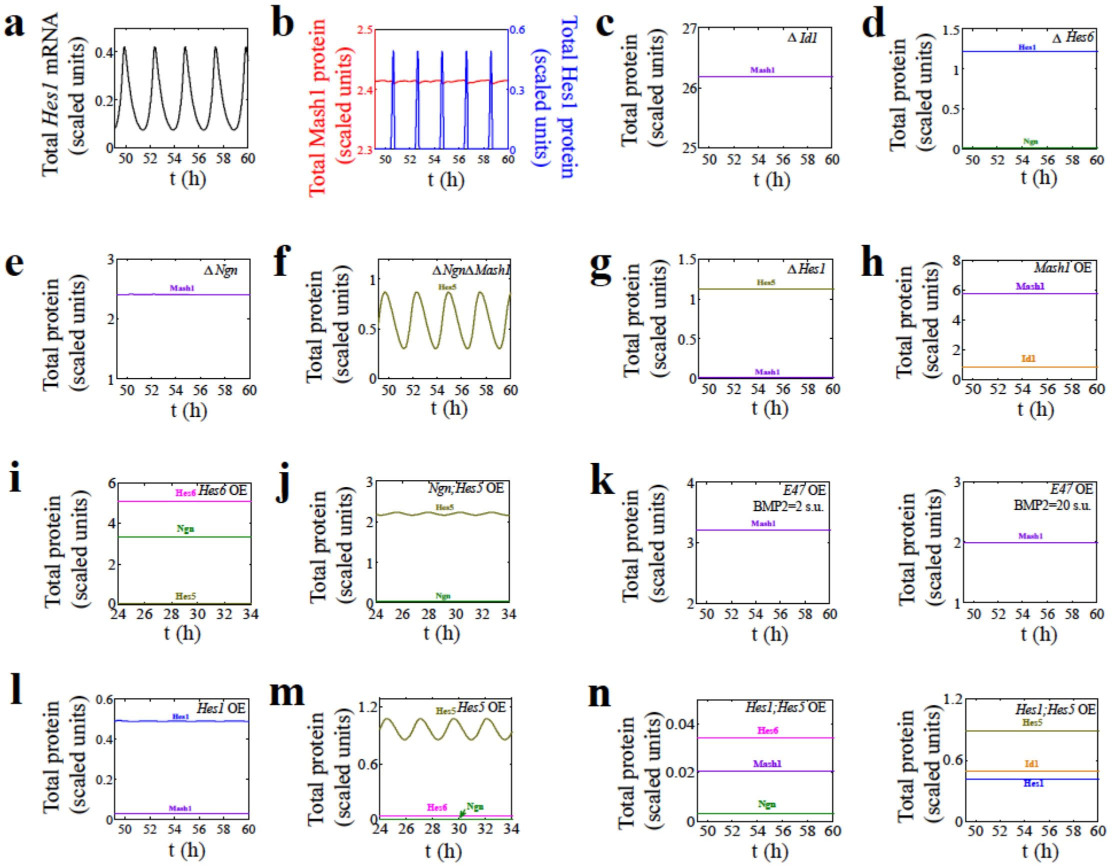

Supplementary Figure 3. Model simulates a number of experimentally observed phenotypes in CNS. **(a)** *Hes1* mRNA shows ~ 2 hr period oscillation at BMP2=20 s.u.. **(b)** Mash1 protein expression oscillates in the opposite phase to Hes1 at BMP2=2 s.u.. **(c)** Deletion of *Id1* gene, G_{idt}=0 s.u., results in the up-regulation of Mash1, which indicates increase in neurogenesis at high dose of BMP2 (BMP2=20 s.u.). **(d)** Deletion of *Hes6* gene (G_{ht}=0 s.u.) favors gliogenesis at low dose of BMP2 (BMP2=2 s.u.). **(e)** Deletion of *Ngn* gene (G_{nt}=0 s.u.) favors gliogenesis at BMP2=2 s.u.. **(f)** *Ngn* and *Hes6* gene double knock-out at BMP2=2 s.u., gliogenesis favored. **(g)** Deletion of *Hes1* gene, G_t=0 s.u., no significant effect at BMP2=20 s.u.. **(h)** Overexpression of *Mash1* gene (G_{mt}=5 X WT) at high BMP2 (BMP2=20 s.u.) results in neurogenesis. **(i)** Overexpression of *Hes6* gene (G_{ht}=5 X WT) at high BMP2 (BMP2=20 s.u.) results in neurogenesis. **(j)** Simultaneous overexpression of *Ngn* and *Hes5* genes (G_{nt}=G_{5t}=5 X WT) predicts the gliogenic fate of the system at high dose of BMP2 (BMP2=20 s.u.) with increase in Hes5 level. **(k)** Overexpression of total *E47* at low and high BMP2 results in neurogenesis. **(l)** Overexpression of *Hes1* gene (G_t=5 X WT) at BMP2=2 s.u., neurogenesis inhibited. **(m)** Overexpression of *Hes5* gene (G_{5t}=5 X WT) at BMP2=2 s.u., gliogenesis favored. **(n)** Simultaneous overexpression of *Hes1* and *Hes5* genes (G_t=G_{5t}=5 X WT) predicts the gliogenic fate of the system at low dose of BMP2 (BMP2=2 s.u.) with decrease in Mash1 level and increase in Hes5 level. Other parameters are same as mentioned in SI Text.

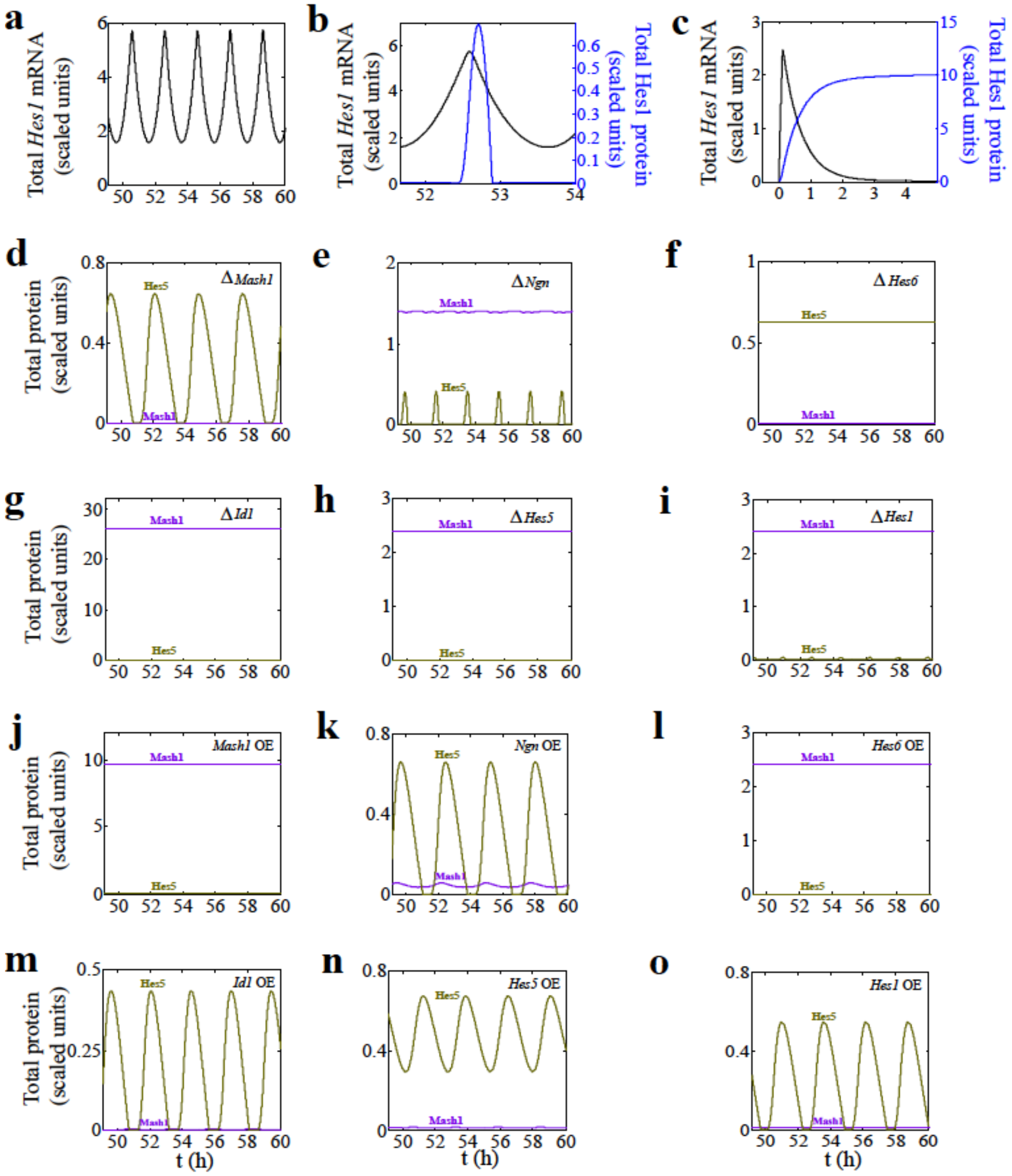

Supplementary Figure 4. Model predicts several mutant and over-expression phenotypes in PNS. **(a)** *Hes1* mRNA shows ~ 2 hr period oscillation. **(b)** Total Hes1 protein oscillation is delayed relative to *Hes1* mRNA oscillation by ~20 minutes. **(c)** Degradation of Hes1 protein is stopped (k_{dhes1}=k_{dyn}=0 min^{-1}) to incorporate the effect of the presence of proteasome inhibitor. This results constant repression of *Hes1* transcription by highly expressing Hes1 protein level. (a-c) Simulations were done at BMP2=20 s.u. **(d)** Deletion of *Mash1* gene (G_{mt}=0 s.u.) results in the up-regulation of Hes5, which indicates inhibition of neurogenesis even at high dose of BMP2 (BMP2=20 s.u.). **(e)** Deletion of *Ngn* gene (G_{nt}=0 s.u.) results in the complementary expression of Mash1 at high dose of BMP2 (BMP2=20 s.u.). **(f)** Deletion of *Hes6* gene (G_{ht}=0 s.u.) results in the up-regulation of Hes5, which indicates inhibition of neurogenesis at high dose of BMP2 (BMP2=20 s.u.). **(g)** Deletion of *Id1* gene (G_{idt}=0 s.u.), **(h)** Deletion of *Hes5* gene (G_{5t}=0 s.u.) and **(i)** Deletion of *Hes1* gene (G_{t}=0 s.u.) result in the up-regulation of Mash1, which indicates increase in neurogenesis at low dose of BMP2 (BMP2=2 s.u.). **(j)** Overexpression of *Mash1* gene (G_{mt}=5 X WT) at low BMP2 (BMP2=2 s.u.) up-regulates Mash1 level resulting in neurogenesis. **(k)** Overexpression of *Ngn* gene (G_{nt}=5 X WT) at low BMP2 (BMP2=2 s.u.), no significant effect. **(l)** Overexpression of *Hes6* gene (G_{ht}=5 X WT) at low BMP2 (BMP2=2 s.u.) up-

regulates Mash1 level and results in neurogenesis. **(m)** Overexpression of *Id1* gene (G_{idt}=5 X WT), **(n)** Overexpression of *Hes5* gene (G_{5t}=5 X WT) and **(o)** Overexpression of *Hes1* gene (G_t=5 X WT) at high BMP2 (BMP2=20 s.u.) result in the down-regulation of Mash1 level and indicate gliogenic fate commitment in all the three cases. In all the figures k_{bmp2}=100 min^{-1} and k_{bmp22}=3e-02 min^{-1} are used. Other parameters are same as depicted in SI Text.

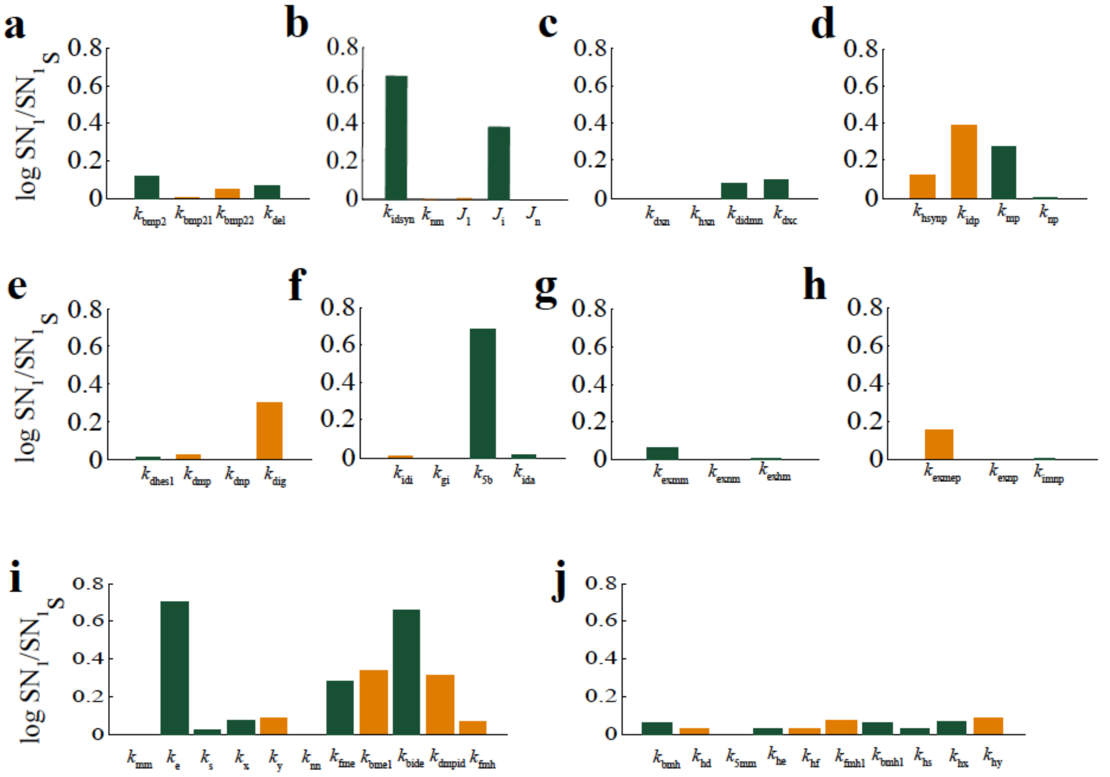

Supplementary Figure 5. Sensitivity analysis of the model parameters by taking the saddle node SN_1 as the sensitivity criteria in CNS. Sensitivity of SN_1 towards different **(a)** BMP2 and Notch related parameters. **(b)** Transcription rates of mRNAs. **(c)** Degradation rates of the mRNAs. **(d)** Translation rates of proteins. **(e)** Degradation rates of the Proteins. **(f)** Activation and inactivation rates of genes. **(g)** Rates of export of mRNAs from cytoplasm. **(h-j)** Rest of the parameters. We were unable to track SN_1 in few cases (as we were unable to locate the position of SN_1 while performing the numerical analysis by using XPPAUT). Orange bar signifies movement of the saddle node (SN_1) towards higher BMP2 and dark green bar signifies movement of the saddle node (SN_1) towards lower BMP2 than the WT CNS case. Parameters are increased individually at an amount of 20% of the model parameters (SI Text) keeping all other parameters constant.

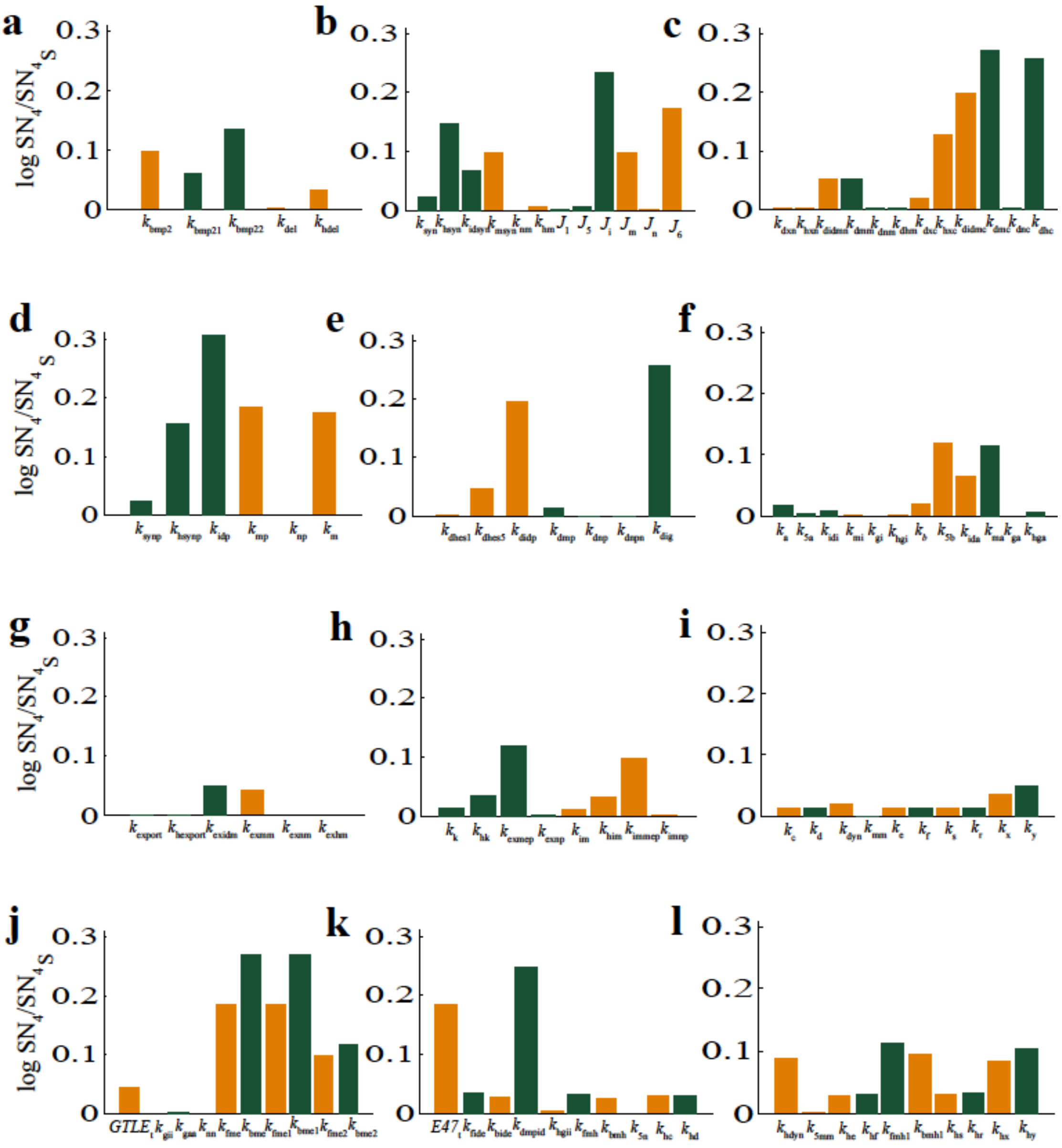

Supplementary Figure 6. Sensitivity analysis of the model parameters by taking the saddle node SN_4 as the sensitivity criteria in CNS. Sensitivity of SN_4 towards different **(a)** BMP2 and Notch related parameters. **(b)** Transcription rates of mRNAs. **(c)** Degradation rates of the mRNAs. **(d)** Translation rates of proteins. **(e)** Degradation rates of the Proteins. **(f)** Activation and inactivation rates of genes. **(g)** Rates of export of mRNAs from cytoplasm. **(h-l)** Rest of the parameters. Orange bar signifies movement of the saddle node (SN_4) towards higher BMP2 and dark green bar signifies movement of the saddle node (SN_4) towards lower BMP2 than the WT CNS case. Parameters are increased individually at an amount of 20% of the model parameters (SI Text) keeping all other parameters constant.

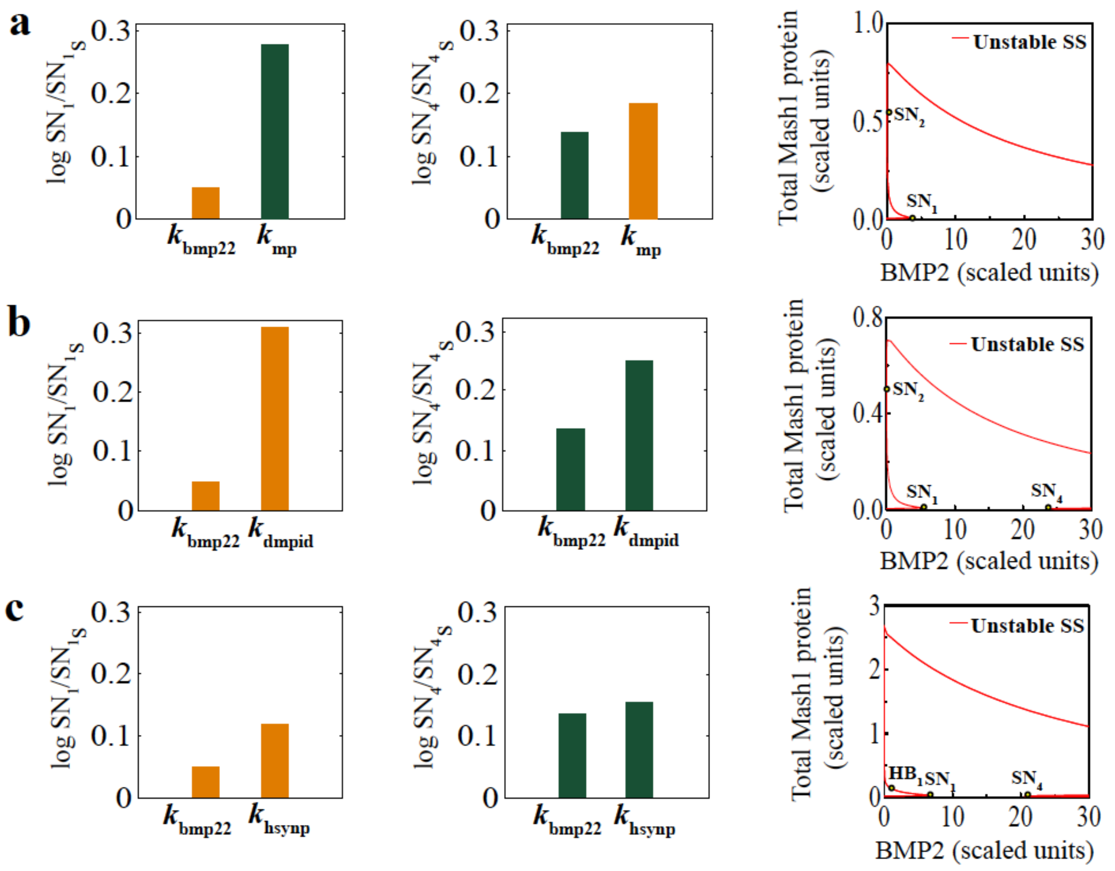

Supplementary Figure 7. Model predicts other possible routes to get PNS like behavior. (a) Left and middle panels show sensitivities of k_{bmp22} and k_{mp} towards SN_1 and SN_4 in CNS. Right panel shows bifurcation diagram of total Mash1 protein as a function of BMP2 at k_{bmp22}=5e-02 min^{-1} and k_{mp}=2.5e-02 min^{-1}. **(b)** Left and middle panels show sensitivities of k_{bmp22} and k_{dmpid} towards SN_1 and SN_4 in CNS. Right panel shows bifurcation diagram of total Mash1 protein as a function of BMP2 at k_{bmp22}=5e-02 min^{-1} and k_{dmpid}=4.5e-01 min^{-1}. **(c)** Left and middle panels show sensitivities of k_{bmp22} and k_{hsynp} towards SN_1 and SN_4 in CNS. Right panel shows bifurcation diagram of total Mash1 protein as a function of BMP2 at k_{bmp22}=4e-02 min^{-1} and k_{hsynp}=1.0 min^{-1}. Orange bar signifies movement of the saddle node towards higher BMP2 and dark green bar signifies movement of the saddle node towards lower BMP2 than the WT CNS case. Both the parameters are increased individually at an amount of 20% of the model parameters (SI Text) keeping all other parameters constant. In all the cases (Figs. S7a-c, right panels) BMP2 induces developmental cell fate change from gliogenic state (low Mash1 expression) to neurogenic state (high Mash1 expression).

SI Text

Equations governing the full model (CNS and PNS)

$$\frac{\mathrm{d}G_a}{\mathrm{d}t} = k_{\mathrm{a}} \cdot (G_t - G_a) - k_{\mathrm{b}} \cdot P_{4\mathrm{n}} \cdot G_a - k_{\mathrm{nn}} \cdot N_{pn} \cdot G_a + k_{\mathrm{del}} \cdot (G_t - G_a) \tag{1}$$

$$\frac{\mathrm{d}H1_{mn}}{\mathrm{d}t} = k_{\mathrm{syn}} \cdot G_a + J_1 \cdot (G_t - G_a) - k_{\mathrm{dxn}} \cdot H1_{mn} - k_{\mathrm{export}} \cdot H1_{mn} \tag{2}$$

$$\frac{\mathrm{d}H1_{mc}}{\mathrm{d}t} = k_{\mathrm{export}} \cdot H1_{mn} - k_{\mathrm{dxc}} \cdot H1_{mc} \tag{3}$$

$$\frac{\mathrm{d}H1_{p2}}{\mathrm{d}t} = k_{\mathrm{c}} \cdot H1_p^2 - k_{\mathrm{d}} \cdot H1_{p2} - k_{\mathrm{e}} \cdot H1_{p2} \cdot (GTLE_t - GP_2 - P_{1C} - P_{2C} - P_{3C} - P_{4C} - P_{4n} - HP_2 - HP_{1C} - HP_{2C} - HP_{3C} - HP_{4C} - HP_{4n}) + k_{\mathrm{f}} \cdot GP_2 \cdot H6_p \tag{4}$$

$$\frac{\mathrm{d}GP_2}{\mathrm{d}t} = k_{\mathrm{e}} \cdot H1_{p2} \cdot (GTLE_t - GP_2 - P_{1C} - P_{2C} - P_{3C} - P_{4C} - P_{4n} - HP_2 - HP_{1C} - HP_{2C} - HP_{3C} - HP_{4C} - HP_{4n}) - k_{\mathrm{f}} \cdot GP_2 \cdot H6_p - k_{\mathrm{s}} \cdot GP_2 + k_{\mathrm{r}} \cdot P_{1C} \tag{5}$$

$$\frac{\mathrm{d}P_{1C}}{\mathrm{d}t} = k_{\mathrm{s}} \cdot GP_2 - k_{\mathrm{r}} \cdot P_{1C} - k_{\mathrm{x}} \cdot P_{1C} + k_{\mathrm{y}} \cdot P_{2C} \tag{6}$$

$$\frac{\mathrm{d}P_{2C}}{\mathrm{d}t} = k_{\mathrm{x}} \cdot P_{1C} - k_{\mathrm{y}} \cdot P_{2C} - k_{\mathrm{x}} \cdot P_{2C} + k_{\mathrm{y}} \cdot P_{3C} \tag{7}$$

$$\frac{\mathrm{d}P_{3C}}{\mathrm{d}t} = k_{\mathrm{x}} \cdot P_{2C} - k_{\mathrm{y}} \cdot P_{3C} - k_{\mathrm{x}} \cdot P_{3C} + k_{\mathrm{y}} \cdot P_{4C} \tag{8}$$

$$\frac{\mathrm{d}P_{4C}}{\mathrm{d}t} = k_{\mathrm{x}} \cdot P_{3C} - k_{\mathrm{y}} \cdot P_{4C} - k_{\mathrm{im}} \cdot P_{4C} + k_{\mathrm{k}} \cdot P_{4n} \tag{9}$$

$$\frac{\mathrm{d}P_{4n}}{\mathrm{d}t} = k_{\mathrm{im}} \cdot P_{4C} - k_{\mathrm{k}} \cdot P_{4n} \tag{10}$$

$$\frac{\mathrm{d}EH_{1C}}{\mathrm{d}t} = k_{\mathrm{fmh}} \cdot H1_p \cdot \left(E47_t - ME - ME_{pc} - 2 \cdot ME_{pc2} - 2 \cdot ME_{pn} - IE - EH_{1C} - EH_{5C}\right) - k_{\mathrm{bmh}} \cdot H6_p \cdot EH_{1C} \tag{11}$$

$$\frac{\mathrm{d}H1_{pt}}{\mathrm{d}t} = k_{\mathrm{synp}} \cdot H1_{mc} - k_{\mathrm{dhes1}} \cdot H1_p - \frac{k_{\mathrm{dyn}} \cdot H1_p \cdot H6_p}{k_{\mathrm{mm}} + H1_p} \tag{12}$$

$$\frac{\mathrm{d}G_{ha}}{\mathrm{d}t} = k_{\mathrm{hgi}} \cdot (G_{ht} - G_{ha}) + k_{\mathrm{hgii}} \cdot (G_{ht} - G_{ha}) \cdot \left(N_{pn} + ME_{pn}\right) - k_{\mathrm{hga}} \cdot G_{ha} \tag{13}$$

$$\frac{\mathrm{d}H6_{mn}}{\mathrm{d}t} = k_{\mathrm{hm}} \cdot G_{ha} + J_6 \cdot (G_{ht} - G_{ha}) - k_{\mathrm{dhm}} \cdot H6_{mn} - k_{\mathrm{exhm}} \cdot H6_{mn} \tag{14}$$

$$\frac{\mathrm{d}H6_{mc}}{\mathrm{d}t} = k_{\mathrm{exhm}} \cdot H6_{mn} - k_{\mathrm{dhc}} \cdot H6_{mc} \tag{15}$$

$$\frac{\mathrm{d}H6_p}{\mathrm{d}t} = k_{\mathrm{m}} \cdot H6_{mc} - k_{\mathrm{dig}} \cdot H6_p \tag{16}$$

$$\frac{dG_{na}}{dt} = k_{\mathrm{gi}} \cdot (G_{nt} - G_{na}) + k_{\mathrm{gii}} \cdot (G_{nt} - G_{na}) \cdot ME_{pn} - k_{\mathrm{ga}} \cdot G_{na} - k_{\mathrm{gaa}} \cdot G_{na} \cdot (P_{4n} + HP_{4n}) \quad (17)$$

$$\frac{dN_m}{dt} = k_{\mathrm{nm}} \cdot G_{na} + J_n \cdot (G_{nt} - G_{na}) - k_{\mathrm{dnm}} \cdot N_m - k_{\mathrm{exnm}} \cdot N_m \quad (18)$$

$$\frac{dN_c}{dt} = k_{\mathrm{exnm}} \cdot N_m - k_{\mathrm{dnc}} \cdot N_c \quad (19)$$

$$\frac{dN_{pn}}{dt} = k_{\mathrm{imnp}} \cdot \left(N_{pt} - N_{pn}\right) - k_{\mathrm{exnp}} \cdot N_{pn} - k_{\mathrm{dnpn}} \cdot N_{pn} \quad (20)$$

$$\frac{dN_{pt}}{dt} = k_{\mathrm{np}} \cdot N_c - k_{\mathrm{dnp}} \cdot \left(N_{pt} - N_{pn}\right) - k_{\mathrm{dnpn}} \cdot N_{pn} \quad (21)$$

$$\frac{dG_{5a}}{dt} = \left(k_{\mathrm{5a}} + k_{\mathrm{bmp22}} \,.\, \mathrm{BMP2}\right) \cdot (G_{5t} - G_{5a}) - k_{\mathrm{5b}} \cdot HP_{4n} \cdot G_{5a} - k_{\mathrm{5n}} \cdot N_{pn} \cdot G_{5a} + k_{\mathrm{hdel}} \cdot (G_{5t} - G_{5a}) \quad (22)$$

$$\frac{dH5_{mn}}{dt} = k_{\mathrm{hsyn}} \cdot G_{5a} + J_5 \cdot (G_{5t} - G_{5a}) - k_{\mathrm{hxn}} \cdot H5_{mn} - k_{\mathrm{hexport}} \cdot H5_{mn} \quad (23)$$

$$\frac{dH5_{mc}}{dt} = k_{\mathrm{hexport}} \cdot H5_{mn} - k_{\mathrm{hxc}} \cdot H5_{mc} \quad (24)$$

$$\frac{dH5_{p2}}{dt} = k_{\mathrm{hc}} \cdot H5_p^2 - k_{\mathrm{hd}} \cdot H5_{p2} - k_{\mathrm{he}} \cdot H5_{p2} \cdot (GTLE_t - GP_2 - P_{1C} - P_{2C} - P_{3C} - P_{4C} - P_{4n} - HP_2 - HP_{1C} - HP_{2C} - HP_{3C} - HP_{4C} - HP_{4n}) + k_{\mathrm{hf}} \cdot HP_2 \cdot H6_p \quad (25)$$

$$\frac{dHP_2}{dt} = k_{\mathrm{he}} \cdot H5_{p2} \cdot (GTLE_t - GP_2 - P_{1C} - P_{2C} - P_{3C} - P_{4C} - P_{4n} - HP_2 - HP_{1C} - HP_{2C} - HP_{3C} - HP_{4C} - HP_{4n}) - k_{\mathrm{hf}} \cdot HP_2 \cdot H6_p - k_{\mathrm{hs}} \cdot HP_2 + k_{\mathrm{hr}} \cdot HP_{1C} \quad (26)$$

$$\frac{dHP_{1C}}{dt} = k_{\mathrm{hs}} \cdot HP_2 - k_{\mathrm{hr}} \cdot HP_{1C} - k_{\mathrm{hx}} \cdot HP_{1C} + k_{\mathrm{hy}} \cdot HP_{2C} \quad (27)$$

$$\frac{dHP_{2C}}{dt} = k_{\mathrm{hx}} \cdot HP_{1C} - k_{\mathrm{hy}} \cdot HP_{2C} - k_{\mathrm{hx}} \cdot HP_{2C} + k_{\mathrm{hy}} \cdot HP_{3C} \quad (28)$$

$$\frac{dHP_{3C}}{dt} = k_{\mathrm{hx}} \cdot HP_{2C} - k_{\mathrm{hy}} \cdot HP_{3C} - k_{\mathrm{hx}} \cdot HP_{3C} + k_{\mathrm{hy}} \cdot HP_{4C} \quad (29)$$

$$\frac{dHP_{4C}}{dt} = k_{\mathrm{hx}} \cdot HP_{3C} - k_{\mathrm{hy}} \cdot HP_{4C} - k_{\mathrm{him}} \cdot HP_{4C} + k_{\mathrm{hk}} \cdot HP_{4n} \quad (30)$$

$$\frac{dHP_{4n}}{dt} = k_{\mathrm{him}} \cdot HP_{4C} - k_{\mathrm{hk}} \cdot HP_{4n} \quad (31)$$

$$\frac{dEH_{5C}}{dt} = k_{\mathrm{fmh1}} \cdot H5_p \cdot \left(E47_t - ME - ME_{pc} - 2 \cdot ME_{pc2} - 2 \cdot ME_{pn} - IE - EH_{1C} - EH_{5C}\right) - k_{\mathrm{bmh1}} \cdot H6_p \cdot EH_{5C} \quad (32)$$

$$\frac{dH5_{pt}}{dt} = k_{\mathrm{hsynp}} \cdot H5_{mc} - k_{\mathrm{dhes5}} \cdot H5_p - \frac{k_{\mathrm{hdyn}} \cdot H5_p \cdot H6_p}{k_{\mathrm{5mm}} + H5_p} \quad (33)$$

$$\frac{dG_{ma}}{dt} = \left(k_{\mathrm{mi}} + k_{\mathrm{bmp2}} . \mathrm{BMP2}\right) \cdot (G_{mt} - G_{ma}) . ME_{pn} - k_{\mathrm{ma}} \cdot G_{ma} \cdot (HP_{4n} + P_{4n}) \quad (34)$$

$$\frac{dM_m}{dt} = k_{\mathrm{msyn}} \cdot G_{ma} + J_{\mathrm{m}} \cdot (G_{mt} - G_{ma}) - k_{\mathrm{dmm}} \cdot M_m - k_{\mathrm{exmm}} \cdot M_m \quad (35)$$

$$\frac{dM_c}{dt} = k_{\mathrm{exmm}} \cdot M_m - k_{\mathrm{dmc}} \cdot M_c \quad (36)$$

$$\frac{dM_p}{dt} = k_{\mathrm{mp}} \cdot M_c - \left(k_{\mathrm{dmp}} + k_{\mathrm{dmpid}} . I_p\right) . M_p - k_{\mathrm{fme}} \cdot M_p \cdot \left(E47_t - ME - ME_{pc} - 2 \cdot ME_{pc2} - 2 \cdot ME_{pn} - IE - EH_{1C} - EH_{5C}\right) + k_{\mathrm{bme}} \cdot ME \quad (37)$$

$$\frac{dME}{dt} = k_{\mathrm{fme}} \cdot M_p \cdot \left(E47_t - ME - ME_{pc} - 2 \cdot ME_{pc2} - 2 \cdot ME_{pn} - IE - EH_{1C} - EH_{5C}\right) - k_{\mathrm{bme}} \cdot ME - k_{\mathrm{fme1}} \cdot ME + k_{\mathrm{bme1}} \cdot ME_{pc} \quad (38)$$

$$\frac{dME_{pc2}}{dt} = k_{\mathrm{fme2}} \cdot ME_{pc}^2 - k_{\mathrm{bme2}} \cdot ME_{pc2} - k_{\mathrm{immep}} \cdot ME_{pc2} + k_{\mathrm{exmep}} \cdot ME_{pn} \quad (39)$$

$$\frac{dME_{pn}}{dt} = k_{\mathrm{immep}} \cdot ME_{pc2} - k_{\mathrm{exmep}} \cdot ME_{pn} \quad (40)$$

$$\frac{dM_t}{dt} = k_{\mathrm{mp}} \cdot M_C - \left(k_{\mathrm{dmp}} + k_{\mathrm{dmpid}} . I_p\right) . M_p \quad (41)$$

$$\frac{dG_{ida}}{dt} = \left(k_{\mathrm{idi}} + k_{\mathrm{bmp21}} . \mathrm{BMP2}\right) \cdot (G_{idt} - G_{ida}) - k_{\mathrm{ida}} \cdot G_{ida} \quad (42)$$

$$\frac{dI_{mn}}{dt} = k_{\mathrm{idsyn}} \cdot G_{ida} + J_{\mathrm{i}} \cdot (G_{idt} - G_{ida}) - k_{\mathrm{didmn}} \cdot I_{mn} - k_{\mathrm{exidm}} \cdot I_{mn} \quad (43)$$

$$\frac{dI_{mc}}{dt} = k_{\mathrm{exidm}} \cdot I_{mn} - k_{\mathrm{didmc}} \cdot I_{mc} \quad (44)$$

$$\frac{d\mathrm{IE}}{dt} = k_{\mathrm{fide}} \cdot I_p \cdot \left(E47_t - ME - ME_{pc} - 2 \cdot ME_{pc2} - 2 \cdot ME_{pn} - IE - EH_{1C} - EH_{5C}\right) - k_{\mathrm{bide}} \cdot IE \quad (45)$$

$$\frac{dI_t}{dt} = k_{idp} \cdot I_{mc} - k_{\mathrm{didp}} \cdot I_p \quad (46)$$

$$ME_{pc} = \left(M_t - M_p - ME - 2 \cdot ME_{pc2} - 2 \cdot ME_{pn}\right) \quad (47)$$

$$I_p = I_t - IE \quad (48)$$

$$H1_p = \left(H1_{pt} - 2 \cdot H1_{p2} - 2 \cdot GP_2 - 2 \cdot P_{1C} - 2 \cdot P_{2C} - 2 \cdot P_{3C} - 2 \cdot P_{4C} - 2 \cdot P_{4n} - EH_{1C}\right) \quad (49)$$

$$H5_p = \left(H5_{pt} - 2 \cdot H5_{p2} - 2 \cdot HP_2 - 2 \cdot HP_{1C} - 2 \cdot HP_{2C} - 2 \cdot HP_{3C} - 2 \cdot HP_{4C} - 2 \cdot HP_{4n} - EH_{5C}\right) \quad (50)$$

Abbreviated names of species in the model and their description	
G_i	Inactive *Hes1* gene
G_a	Active *Hes1* gene
G_t	Total *Hes1* gene
$H1_{mn}$	*Hes1* mRNA in nucleus
$H1_{mc}$	*Hes1* mRNA in cytoplasm
$H1_p$	Hes1 protein
$H1_{p2}$	Hes1 protein dimer
GP_2	$H1_{p2}$:GroTLE
P_{1c}	Phosphorylated form of GP_2 in cytoplasm
P_{2c}	Doubly phosphorylated form of GP_2 in cytoplasm
P_{3c}	Triply phosphorylated form of GP_2 in cytoplasm
P_{4c}	Four times phosphorylated form of GP_2 in cytoplasm
P_{4n}	Four times phosphorylated form of GP_2 in nucleus
EH_{1c}	$H1_p$:E47
$H1_{pt}$	Total Hes1 protein
G_{hi}	Inactive *Hes6* gene
G_{ha}	Active *Hes6* gene
G_{ht}	Total *Hes6* gene
$H6_{mn}$	*Hes6* mRNA in nucleus
$H6_{mc}$	*Hes6* mRNA in cytoplasm
$H6_p$	Hes6 protein
G_{ni}	Inactive *Ngn* gene
G_{na}	Active *Ngn* gene
G_{nt}	Total *Ngn* gene
N_m	*Ngn* mRNA in nucleus
N_c	*Ngn* mRNA in cytoplasm
N_{pc}	Ngn protein in cytoplasm
N_{pn}	Ngn protein in nucleus
N_{pt}	Total Ngn protein
G_{5i}	Inactive *Hes5* gene
G_{5a}	Active *Hes5* gene
G_{5t}	Total *Hes5* gene
$H5_{mn}$	*Hes5* mRNA in nucleus
$H5_{mc}$	*Hes5* mRNA in cytoplasm
$H5_p$	Hes5 protein
$H5_{p2}$	Hes5 protein dimer
HP_2	$H5_{p2}$:GroTLE

HP_{1c}	Phosphorylated form of HP_2 in cytoplasm
HP_{2c}	Doubly phosphorylated form of HP_2 in cytoplasm
HP_{3c}	Triply phosphorylated form of HP_2 in cytoplasm
HP_{4c}	Four times phosphorylated form of HP_2 in cytoplasm
HP_{4n}	Four times phosphorylated form of HP_2 in nucleus
EH_{5c}	$H5_p$:E47
$H5_{pt}$	Total Hes5 protein
G_{mi}	Inactive *Mash1* gene
G_{ma}	Active *Mash1* gene
G_{mt}	Total *Mash1* gene
M_m	*Mash1* mRNA in nucleus
M_c	*Mash1* mRNA in cytoplasm
M_p	Mash1 protein
ME	M_p:E47
ME_{pc}	Phosphorylated form of ME in cytoplasm
ME_{pc2}	ME_{pc} dimer
ME_{pn}	ME_{pc2} in nucleus
M_t	Total Mash1 protein
G_{idi}	Inactive *Id1* gene
G_{ida}	Active *Id1* gene
G_{idt}	Total *Id1* gene
I_{mn}	*Id1* mRNA in nucleus
I_{mc}	*Id1* mRNA in cytoplasm
I_p	Id1 protein
IE	I_p:E47
I_t	Total Id1 protein
$E47_t$	Total E47 protein
E47	Free E47 protein
$GTLE_t$	Total Gro/TLE protein
GroTLE	Free Gro/TLE protein
BMP2	Bone morphogenetic protein 2

Description of the parameters, their values and sources for CNS		
**Description**	**Symbol**	**Value**
Degradation rates of *Hes1, Hes6, Hes5, Ngn*, *Mash1*, *Id1* mRNA's in nucleus and cytoplasm respectively[15,27,28] (min^{-1})	k_{dxn}, k_{dxc}, k_{dhm}, k_{dhc}, k_{hxn}, k_{hxc}, k_{dnm}, k_{dnc}, k_{dmm}, k_{dmc}, k_{didmn}, k_{didmc}	0.0288
Degradation rate of Hes1 Protein[15,27,28] (min^{-1})	k_{dhes1}	0.031
Degradation rate of Hes6 Protein[28] (min^{-1})	k_{dig}	0.031
Degradation rate of Ngn Protein in cytoplasm and nucleus[29] (min^{-1})	k_{dnp}, k_{dnpn}	0.031
Degradation rate of Hes5 Protein (min^{-1})	k_{dhes5}	0.031
Degradation rate of Mash1 Protein[30] (min^{-1})	k_{dmp}	0.003
Degradation rate of Id1 Protein[31] (min^{-1})	k_{didp}	0.023
Rate of translation from *Hes1* mRNA (min^{-1})	k_{synp}	0.1
Rate of translation from *Hes6* mRNA (min^{-1})	k_{m}	0.1
Rate of translation from *Ngn* mRNA (min^{-1})	k_{np}	0.01
Rate of translation from *Hes5* mRNA (min^{-1})	k_{hsynp}	0.08
Rate of translation from *Mash1* mRNA (min^{-1})	k_{mp}	9.47X10^{-2}
Rate of translation from *Id1* mRNA (min^{-1})	k_{idp}	1.0
Transcriptional rate for *Hes1* mRNA from inactive gene (min^{-1})	J_1	0.3
Transcriptional rate for *Hes6* mRNA from inactive gene (min^{-1})	J_6	0.3
Transcriptional rate for *Ngn* mRNA from inactive gene (min^{-1})	J_n	0.3
Transcriptional rate for *Hes5* mRNA from inactive gene (min^{-1})	J_5	0.3
Transcriptional rate for *Mash1* mRNA from inactive gene (min^{-1})	J_m	0.3
Transcriptional rate for *Id1* mRNA from inactive gene (min^{-1})	J_i	0.3
Transcriptional rate for *Hes1* mRNA from active gene (min^{-1})	k_{syn}	390.0
Transcriptional rate for *Hes6* mRNA from active gene (min^{-1})	k_{hm}	300.0
Transcriptional rate for *Ngn* mRNA from active gene (min^{-1})	k_{nm}	300.0
Transcriptional rate for *Hes5* mRNA from active gene (min^{-1})	k_{hsyn}	253.5
Transcriptional rate for *Mash1* mRNA from active gene (min^{-1})	k_{msyn}	29.62
Transcriptional rate for *Id1* mRNA from active gene (min^{-1})	k_{idsyn}	43.838
Hes1 gene activation rate (min^{-1})	k_a	2.0
Hes6 gene activation rate (min^{-1})	k_{hgi}	0.01
Ngn gene activation rate (min^{-1})	k_{gi}	1X10^{-3}
Hes5 gene activation rate (min^{-1})	k_{5a}	2.0

Mash1 gene activation rate (min^{-1})	k_{mi}	30.0
Id1 gene activation rate (min^{-1})	k_{idi}	0.02
Hes1 gene inactivation rate (min^{-1})	k_{b}	$8X10^{6}$
Hes6 gene inactivation rate (min^{-1})	k_{hga}	$1X10^{3}$
Ngn gene inactivation rate (min^{-1})	k_{ga}	$1X10^{-3}$
Hes5 gene inactivation rate (min^{-1})	k_{5b}	$8X10^{6}$
Mash1 gene inactivation rate (min^{-1})	k_{ma}	$81.31X10^{3}$
Id1 gene inactivation rate (min^{-1})	k_{ida}	81.31
Nuclear export rate of *Hes1* mRNA (min^{-1})	k_{export}	7.15
Nuclear export rate of *Hes6* mRNA (min^{-1})	k_{exhm}	5.5
Nuclear export rate of *Ngn* mRNA (min^{-1})	k_{exnm}	11.0
Nuclear export rate of *Hes5* mRNA (min^{-1})	$k_{hexport}$	4.6475
Nuclear export rate of *Mash1* mRNA (min^{-1})	k_{exmm}	0.1
Nuclear export rate of *Id1* mRNA (min^{-1})	k_{exidm}	0.1
Nuclear export rate of P_{4n} (min^{-1})	k_{k}	0.8
Nuclear export rate of N_{pn} (min^{-1})	k_{exnp}	8.0
Nuclear export rate of HP_{4n} (min^{-1})	k_{hk}	0.8
Nuclear export rate of ME_{pn} (min^{-1})	k_{exmep}	1.0
Nuclear import rate of P_{4C} (min^{-1})	k_{im}	0.1
Nuclear import rate of N_{pc} (min^{-1})	k_{imnp}	0.01
Nuclear import rate of HP_{4C} (min^{-1})	k_{him}	0.1
Nuclear import rate of ME_{pc2} (min^{-1})	k_{immep}	0.8
Hes1 protein and E47 protein association constant (min^{-1})	k_{fmh}	100.0
Hes5 protein and E47 protein association constant (min^{-1})	k_{fmh1}	100.0
Mash1 protein and E47 protein association constant (min^{-1})	k_{fme}	30.0
Id1 protein and E47 protein association constant (min^{-1})	k_{fide}	100.0
EH_{1C} dissociation constant (min^{-1})	k_{bmh}	300.0
EH_{5C} dissociation constant (min^{-1})	k_{bmh1}	300.0
ME dissociation constant (min^{-1})	k_{bme}	81.31
IE dissociation constant (min^{-1})	k_{bide}	81.31
Hes1 protein dimerization constant (min^{-1})	k_{c}	160.0
Hes5 protein dimerization constant (min^{-1})	k_{hc}	128.0
ME_{pc} dimerization constant (min^{-1})	k_{fme2}	400.0
$H1_{p2}$ dissociation constant (min^{-1})	k_{d}	640.0
$H5_{p2}$ dissociation constant (min^{-1})	k_{hd}	512.0
ME_{pc2} dissociation constant (min^{-1})	k_{bme2}	81.31
Gro/TLE protein association constants with $H1_{p2}$ and $H5_{p2}$ (min^{-1})	k_{e} , k_{he}	100.0
GP_2 and HP_2 dissociation constants (min^{-1})	k_{f} , k_{hf}	$1X10^{3}$
Phosphorylation rates of GP_2 and HP_2 (min^{-1})	k_{s} , k_{hs}	0.1

Phosphorylation rate of P_{1C}, P_{2C}, P_{3C} (min^{-1})	k_x	0.8
Phosphorylation rate of HP_{1C}, HP_{2C}, HP_{3C} (min^{-1})	k_{hx}	0.8
Phosphorylation rate of ME (min^{-1})	k_{fme1}	1.0
Dephosphorylation rates of P_{1C} and HP_{1C} (min^{-1})	k_r , k_{hr}	1.0
Dephosphorylation rate of P_{2C}, P_{3C}, P_{4C} (min^{-1})	k_y	0.6
Dephosphorylation rate of HP_{2C}, HP_{3C}, HP_{4C} (min^{-1})	k_{hy}	0.6
Dephosphorylation rate of ME_{pc} (min^{-1})	k_{bme1}	1.0
Repression constants of N_{pn} on *Hes1* and *Hes5* gene activation (min^{-1})	k_{nn} , k_{5n}	1.0
Degradation constants (min^{-1}) of Hes1 Protein facilitated by $H6_p$ and Michaelis constant (s.u.)	k_{dyn} , k_{mm}	0.5, $1X10^{-5}$
Degradation constants (min^{-1}) of Hes5 Protein facilitated by $H6_p$ and Michaelis constant (s.u.)	k_{hdyn} , k_{5mm}	0.4, $1X10^{-5}$
Rate constant of Mash1 and Ngn Protein facilitated *Hes6* gene activation (min^{-1})	k_{hgii}	600.0
Rate constant of *Ngn* gene activation promoted by ME_{pn} (min^{-1})	k_{gii}	200.0
Repression constant of P_{4n} and HP_{4n} on *Ngn* gene activation (min^{-1})	k_{gaa}	$1X10^6$
Degradation rate of M_p facilitated by I_p (min^{-1})	k_{dmpid}	0.1
Total gene of *Hes1, Hes6, Hes5, Ngn*, *Mash1*, *Id1* respectively (s.u.)	G_t , G_{ht} , G_{5t} , G_{nt} , G_{mt} , G_{idt}	0.001
Total E47 protein (s.u.)	$E47_t$	1.0
Total Gro/TLE protein (s.u.)	$GTLE_t$	0.4
Rate constants of Notch activation for *Hes1* and *Hes5* (min^{-1})	k_{del} , k_{hdel}	1.0
Rate constant related to the effect of BMP2 on *Mash1* gene activation (min^{-1})	k_{bmp2}	1900.0
Rate constant related to the effect of BMP2 on *Id1* gene activation (min^{-1})	k_{bmp21}	$3X10^{-2}$
Rate constant related to the effect of BMP2 on *Hes5* gene activation (min^{-1})	k_{bmp22}	12.0

For module-1 (*SI Appendix*, Fig. A1) we use k_{bmp21}=6e-02 min^{-1}. Other parameters are same as above-mentioned parameters.

SI Appendix

Detailed description of Model construction

The model, schematized in Fig. 1 and Supplementary Fig. 1, consists of three modules. Modules 1-3 are composed and combined through various regulatory interactions in a mechanistic fashion. The equations are solely based on mass action kinetics with only two Michaelis-Menten kinds of phenomenological terms. The entire gene regulatory network (GRN) (Supplementary Fig. 1) is constructed based on available experimental literatures. Module-1 contains the Mash1, Id1 and E47 proteins where BMP2 regulates both Mash1 and Id1 at the transcriptional level. We systematically construct and analyze the module-1 first and then added the module-2 to this module-1. Module-2 consists of gene regulatory interactions of Neurogenin (Ngn), Hes6 and Hes5 where BMP2 again activates Hes5 transcriptionally. These two modules are further coupled and extended by adding *Hes1* gene regulatory dynamics (module-3) on it.

Construction of module-1

Mash1 plays an important role in lineage specific cell fate determination of neural stem cells (NSC's) in both CNS and PNS[1–5]. Mash1 hetero-dimerizes with ubiquitously expressed bHLH protein E47 via their HLH domain[1,2,19,25]. This heterodimer formation induces CK2 mediated phosphorylation of Mash1 on Ser^{152}, which further increases heterodimer interaction[2]. After subsequent phosphorylation of Mash1, the phosphorylated heteromeric complex binds to promoter regions of genes containing a CANNTG sequence (E box) in DNA through its basic domain and in the process activates the transcriptional event of Mash1 in a positive feedback manner[1,2,4,19]. This basic region is absent in Id1 proteins, which is one of the subfamilies of HLH-factors[1]. Experimental evidences suggest that Mash1 promotes its own expression by reinforcing responsiveness to BMP2 through a positive-feedback loop[4,28]. BMP2 is responsible for maintenance of the expression of Mash1[4]. This BMP2 driven self-inducing positive feedback loop of Mash1 promotes neuronal fate commitment in neural crest stem cells[1,3–5]. Also BMP2 stimulation is known to up regulate Id1 expression[1]. Activation of BMP2 leads to an increase in the

expression level of Id1[1]. Id1 antagonizes Mash1 in two ways. Id1 competitively binds with E47 protein by sequestering E47 protein (corresponds the Id1 to E47 repression link in Module-1 of Fig. 1 (main text)) away from Mash1, which leads to the formation of transcriptionally inactive complexes and also it induces degradation (corresponds the Id1 to Mash1 repression link in Module-1 of Fig. 1 (main text)) of Mash1 monomer[2]. Mash1 protein stability is critically governed by E47/Id1 expression ratio[2].

Keeping all the observations in mind module-1 (Fig. A1a) is constructed where we consider BMP2 stimulates both *Mash1* and *Id1* gene expressions at k_{bmp2} and k_{bmp21} rates respectively (Supplementary Fig. 1). *Mash1* mRNA (M_m) is transported from nucleus to cytoplasm. In cytoplasm *Mash1* mRNA is designated as M_C. Mash1 protein (M_p) forms heterodimer (ME) with E47. In ME, Mash1 undergoes CK2 mediated phosphorylation. The effect of CK2 mediated phosphorylation is incorporated in the rate constant k_{fme1}. The phosphorylated form (ME_{pc}) forms dimer ME_{pc2}. ME_{pc2} gets transported in to the nucleus and in the model it is abbreviated as ME_{pn}, which contributes to the *Mash1* gene activation (Supplementary Fig. 1). This implies that Mash1 auto-regulates its own expression in presence of BMP2. Similarly *Id1* mRNA (I_{mn}) is transported from nucleus to cytoplasm. In cytoplasm Id1 protein (I_p) is translated from its mRNA (I_{mc}). I_p sequesters E47 by making an inactive complex (IE) (corresponds the Id1 to E47 repression link in Module-1 of Fig. 1 (main text)). I_p also activates the degradation of M_p at k_{dmpid} rate (Supplementary Fig. 1) (corresponds the Id1 to Mash1 repression link in Module-1 of Fig. 1 (main text)). Thus Id1 negatively regulates Mash1 expression in two possible pathways mentioned above. The equations for module-1 (Fig. A1a) are given in A1 Table (parameters values used are given in SI Text).

A1 Table: Equations governing the module-1

Equation	No.
$\frac{dG_{ma}}{dt} = (k_{\text{mi}} + k_{\text{bmp2}}.\text{BMP2}) \cdot (G_{mt} - G_{ma}).ME_{pn} - k_{\text{ma}} \cdot G_{ma}$	1
$\frac{dM_m}{dt} = k_{\text{msyn}} \cdot G_{ma} + J_{\text{m}} \cdot (G_{mt} - G_{ma}) - k_{\text{dmm}} \cdot M_m - k_{\text{exmm}} \cdot M_m$	2
$\frac{dM_c}{dt} = k_{\text{exmm}} \cdot M_m - k_{\text{dmc}} \cdot M_c$	3

$$\frac{dM_p}{dt} = k_{mp} \cdot M_c - (k_{dmp} + k_{dmpid} . I_p). M_p - k_{fme} \cdot M_p \cdot (E47_t - ME - ME_{pc} - 2 \cdot ME_{pc2} - 2 \cdot ME_{pn} - IE) + k_{bme} \cdot ME \quad (4)$$

$$\frac{dME}{dt} = k_{fme} \cdot M_p \cdot (E47_t - ME - ME_{pc} - 2 \cdot ME_{pc2} - 2 \cdot ME_{pn} - IE) - k_{bme} \cdot ME - k_{fme1} \cdot ME + k_{bme1} \cdot ME_{pc} \quad (5)$$

$$\frac{dME_{pc2}}{dt} = k_{fme2} \cdot ME_{pc}^2 - k_{bme2} \cdot ME_{pc2} - k_{immep} \cdot ME_{pc2} + k_{exmep} \cdot ME_{pn} \quad (6)$$

$$\frac{dME_{pn}}{dt} = k_{immep} \cdot ME_{pc2} - k_{exmep} \cdot ME_{pn} \quad (7)$$

$$\frac{dM_t}{dt} = k_{mp} \cdot M_C - (k_{dmp} + k_{dmpid} . I_p). M_p \quad (8)$$

$$\frac{dG_{ida}}{dt} = (k_{idi} + k_{bmp21} . \mathrm{BMP2}) \cdot (G_{idt} - G_{ida}) - k_{ida} \cdot G_{ida} \quad (9)$$

$$\frac{dI_{mn}}{dt} = k_{idsyn} \cdot G_{ida} + J_i \cdot (G_{idt} - G_{ida}) - k_{didmn} \cdot I_{mn} - k_{exidm} \cdot I_{mn} \quad (10)$$

$$\frac{dI_{mc}}{dt} = k_{exidm} \cdot I_{mn} - k_{didmc} \cdot I_{mc} \quad (11)$$

$$\frac{dIE}{dt} = k_{fide} \cdot I_p \cdot (E47_t - ME - ME_{pc} - 2 \cdot ME_{pc2} - 2 \cdot ME_{pn} - IE) - k_{bide} \cdot IE \quad (12)$$

$$\frac{dI_t}{dt} = k_{idp} \cdot I_{mc} - k_{didp} \cdot I_p \quad (13)$$

$$ME_{pc} = (M_t - M_p - ME - 2 \cdot ME_{pc2} - 2 \cdot ME_{pn}) \quad (14)$$

$$I_p = I_t - IE \quad (15)$$

The bifurcation diagram provided in Fig. A1b, interestingly, contains two interconnected bi-stable region in the steady state level of total Mash1 protein as a function of BMP2. From experiments it is well known that one can designate a low Mash1 expressing state as gliogenic like state and a high Mash1 expressing state as neurogenic like state[1-5]. The possible reason behind the first bi-stable region can be attributed to the fact that with increase in BMP2 dose, Mash1 positive feedback on its own expression reinforces the system to switch from a gliogenic to neurogenic like state. By the time the system gets into higher level of Mash1, there is enough accumulation of Id1 protein due to increase in BMP2 level. Elevated level of Id1 finally starts to play its inhibitory role towards Mash1 by sequestering E47 more dominantly and through degradation of Mash1 protein (M_p). Thus positive feedback

of Mash1 in presence of BMP2 on its own synthesis decreases with increase in BMP2 in CNS. This affects the dynamics of the system by producing another bi-stable switch this time from a neurogenic like state (high Mash1 level) to a gliogenic like state (Low level of Mash1) with increase in BMP2 doses. This indicates that module-1 has the potential to show both a CNS and PNS like features simultaneously but the differential nature of NSC's are not observable for a fixed low and high values of BMP2 and also the system does not show any oscillations in Mash1 dynamics as well.

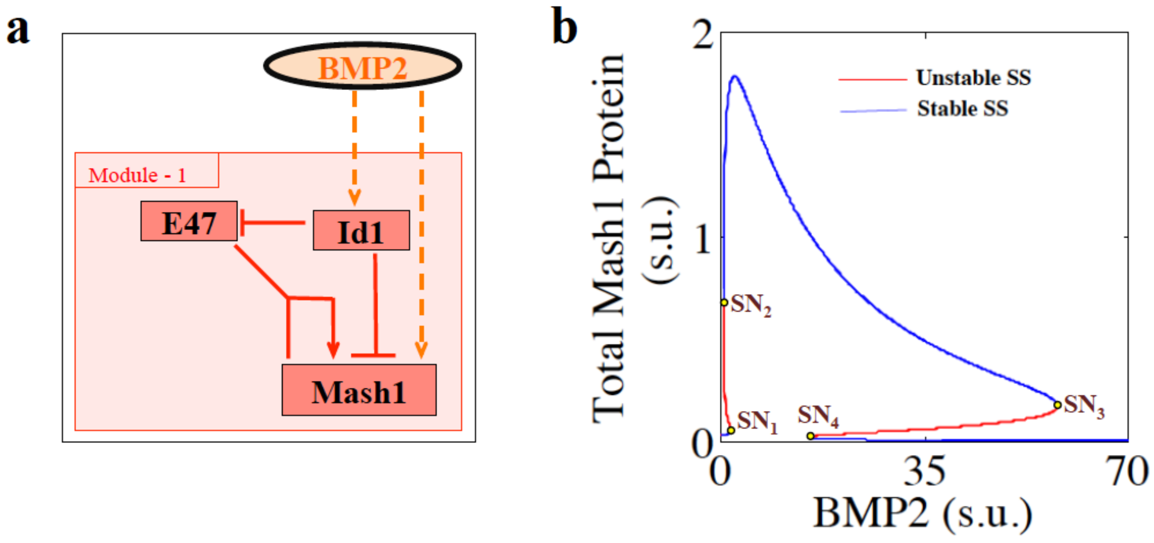

Figure A1. (a) Schematic representation of module-1. **(b)** We plot steady state level of total Mash1 protein as a function of BMP2 in module-1. Parameters are given in SI Text.

Extending module-1 by incorporating module-2

Fig. A2a depicts the core regulatory network contained in module-2 (for molecular details see Supplementary Fig. 1). BMP2 promotes the expression of negative HLH factor Hes5[1]. Hes5 expression is also induced by Notch stimulation[1,7,20,26]. Hes5 protein dimer forms complex with Groucho/transducin-like Enhancer of split (Gro/TLE) proteins and subsequent hyper-phosphorylation of Gro/TLE takes place in the complex[8,21-24,26]. This kind of phosphorylated complex translocates into the nucleus and binds with the promoter region of Hes5 as well as other pro-neural genes (for example, Ngn) to repress (corresponds the self repression link of Hes5 as well as Hes5 to Ngn repression link in Module-2 of Fig. 1 (main text)) their transcription[6,8,11,21-24,26]. Another member of Hes family termed Hes6 inhibits (corresponds the Hes6 to Hes5 repression link in Module-2 of Fig. 1 (main text)) the complex formation of Hes5 and Gro/TLE[7,12]. Thus Hes6 reduces the capability of Hes5 to repress the transcriptional activity of Ngn, thereby promoting neurogenesis.

Hes6 also promotes proteolytic degradation (corresponds the Hes6 to Hes5 repression link in Module-2 of Fig. 1 (main text)) of Hes5 protein similar to Hes1[7,12]. Hes5 forms non-functional heterodimer with E47 and inhibits (corresponds the Hes5 to E47 repression link between Module-2 to Module-1 of Fig. 1 (main text)) its functional activity[1]. We assume Hes6 suppresses Hes5 to form complex with E47 similar to Hes1[25]. Pro-neural protein Ngn positively regulates Hes6 expression in committed neuronal precursors[6,7]. Pro-neural genes not only promote neuronal differentiation but also inhibit astrocyte-specific gene expression[11]. Therefore, our model also accounts for the transcriptional repression of Hes5 gene by Ngn (Details are shown in Supplementary Fig. 1).

We consider *Hes5* transcription occurs in nucleus from both inactive gene (G_{5i}) and active gene (G_{5a}). *Hes5* mRNA ($H5_{mn}$) is transported from nucleus to cytoplasm. In cytoplasm Hes5 protein ($H5_p$) is translated from *Hes5* mRNA ($H5_{mc}$) in cytoplasm. $H5_p$ forms a dimer ($H5_{p2}$). $H5_{p2}$ binds with another protein Gro/TLE and forms a complex (HP_2). Then hyper-phosphorylation of Gro/TLE takes place in the complex. We consider four times phosphorylation of Gro/TLE in HP_2. Then the hyper-phosphorylated complex is transported to the nucleus. It is termed as HP_{4n}. Hes5 represses its own transcription (corresponds the self repression link of Hes5 in Module-2 of Fig. 1 (main text)) through HP_{4n} similarly as Hes1. HP_{4n} also inhibits (corresponds the Hes5 to Ngn repression link in Module-2 of Fig. 1 (main text)) *Ngn* gene activation. *Ngn* mRNA (N_m) is transported from nucleus to cytoplasm. In cytoplasm Ngn protein (N_{pc}) is translated from *Ngn* mRNA (N_c). N_{pc} is transported from cytoplasm to nucleus. The Ngn protein in nucleus is labeled as N_{pn}. N_{pn} promotes *Hes6* gene activation and inhibits (corresponds the Ngn to Hes5 repression link in Module-2 of Fig. 1 (main text)) *Hes5* gene activation. *Hes6* mRNA ($H6_{mn}$) is transported from nucleus to cytoplasm. In cytoplasm Hes6 protein ($H6_p$) is translated from *Hes6* mRNA ($H6_{mc}$). $H6_p$ inhibits the formation of HP_2. We consider $H6_p$ also induces the degradation (corresponds the Hes6 to Hes5 repression link in Module-2 of Fig. 1 (main text)) of $H5_p$ similarly as it does in case of Hes1. $H5_p$ forms a non-functional dimer (EH_{5C}) with E47. $H6_p$ inhibits Hes5 to form EH_{5C}. We consider BMP2 stimulates *Hes5* gene expression at k_{bmp22} rate. The effect of Notch stimulation on *Hes5* is incorporated in the rate constant k_{hdel}.

After constructing the gene interaction network for module-2, we couple module-2 with module-1 with the following additional interactions. ME_{pn} induces gene activation of *Ngn* and *Hes6*, whereas HP_{4n} suppresses (corresponds the Hes5 to Mash1 repression link between Module-2 to Module-1 of Fig. 1 (main text)) gene activation of *Mash1*. The equations for the extended model (Fig. A2a) are given in A2 Table (parameters are given in SI Text). All the interactions shown in module-1 and module-2 are modeled as mass-action kinetic terms as shown in A2 Table except the Hes6 mediated degradation of Hes5.

A2 Table: Equations governing the extended model (module-1+ module-2 with additional interactions)

$\frac{dG_{ha}}{dt} = k_{\mathrm{hgi}} \cdot (G_{ht} - G_{ha}) + k_{\mathrm{hgii}} \cdot (G_{ht} - G_{ha}) \cdot (N_{pn} + ME_{pn}) - k_{\mathrm{hga}} \cdot G_{ha}$	1
$\frac{dH6_{mn}}{dt} = k_{\mathrm{hm}} \cdot G_{ha} - k_{\mathrm{dhm}} \cdot H6_{mn} - k_{\mathrm{exhm}} \cdot H6_{mn} + J_6 \cdot (G_{ht} - G_{ha})$	2
$\frac{dH6_{mc}}{dt} = k_{\mathrm{exhm}} \cdot H6_{mn} - k_{\mathrm{dhc}} \cdot H6_{mc}$	3
$\frac{dH6_{p}}{dt} = k_{\mathrm{m}} \cdot H6_{mc} - k_{\mathrm{dig}} \cdot H6_{p}$	4
$\frac{dG_{na}}{dt} = k_{\mathrm{gi}} \cdot (G_{nt} - G_{na}) + k_{\mathrm{gii}} \cdot (G_{nt} - G_{na}) \cdot ME_{pn} - k_{\mathrm{ga}} \cdot G_{na} - k_{\mathrm{gaa}} \cdot G_{na} \cdot HP_{4n}$	5
$\frac{dN_{m}}{dt} = k_{\mathrm{nm}} \cdot G_{na} - k_{\mathrm{dnm}} \cdot N_{m} - k_{\mathrm{exnm}} \cdot N_{m} + J_n \cdot (G_{nt} - G_{na})$	6
$\frac{dN_{c}}{dt} = k_{\mathrm{exnm}} \cdot N_{m} - k_{\mathrm{dnc}} \cdot N_{c}$	7
$\frac{dN_{pn}}{dt} = k_{\mathrm{imnp}} \cdot (N_{pt} - N_{pn}) - k_{\mathrm{exnp}} \cdot N_{pn} - k_{\mathrm{dnpn}} \cdot N_{pn}$	8
$\frac{dN_{pt}}{dt} = k_{\mathrm{np}} \cdot N_{c} - k_{\mathrm{dnp}} \cdot (N_{pt} - N_{pn}) - k_{\mathrm{dnpn}} \cdot N_{pn}$	9
$\frac{dG_{5a}}{dt} = (k_{\mathrm{5a}} + k_{\mathrm{bmp22}} . \mathrm{BMP2}) \cdot (G_{5t} - G_{5a}) - k_{\mathrm{5b}} \cdot HP_{4n} \cdot G_{5a} - k_{\mathrm{5n}} \cdot N_{pn} \cdot G_{5a} + k_{\mathrm{hdel}} \cdot (G_{5t} - G_{5a})$	10
$\frac{dH5_{mn}}{dt} = k_{\mathrm{hsyn}} \cdot G_{5a} - k_{\mathrm{hexport}} \cdot H5_{mn} - k_{\mathrm{hxn}} \cdot H5_{mn} + J_5 \cdot (G_{5t} - G_{5a})$	11
$\frac{dH5_{mc}}{dt} = k_{\mathrm{hexport}} \cdot H5_{mn} - k_{\mathrm{hxc}} \cdot H5_{mc}$	12

$$\frac{dH5_{p2}}{dt} = k_{\mathrm{hc}} \cdot H5_p^2 - k_{\mathrm{hd}} \cdot H5_{p2} - k_{\mathrm{he}} \cdot H5_{p2} \cdot (GTLE_t - HP_2 - HP_{1C} - HP_{2C} - HP_{3C} - HP_{4C} - HP_{4n}) + k_{\mathrm{hf}} \cdot HP_2 \cdot H6_p \tag{13}$$

$$\frac{dHP_2}{dt} = k_{\mathrm{he}} \cdot H5_{p2} \cdot (GTLE_t - HP_2 - HP_{1C} - HP_{2C} - HP_{3C} - HP_{4C} - HP_{4n}) - k_{\mathrm{hf}} \cdot HP_2 \cdot H6_p - k_{\mathrm{hs}} \cdot HP_2 + k_{\mathrm{hr}} \cdot HP_{1C} \tag{14}$$

$$\frac{dHP_{1C}}{dt} = k_{\mathrm{hs}} \cdot HP_2 - k_{\mathrm{hr}} \cdot HP_{1C} - k_{\mathrm{hx}} \cdot HP_{1C} + k_{\mathrm{hy}} \cdot HP_{2C} \tag{15}$$

$$\frac{dHP_{2C}}{dt} = k_{\mathrm{hx}} \cdot HP_{1C} - k_{\mathrm{hy}} \cdot HP_{2C} - k_{\mathrm{hx}} \cdot HP_{2C} + k_{\mathrm{hy}} \cdot HP_{3C} \tag{16}$$

$$\frac{dHP_{3C}}{dt} = k_{\mathrm{hx}} \cdot HP_{2C} - k_{\mathrm{hy}} \cdot HP_{3C} - k_{\mathrm{hx}} \cdot HP_{3C} + k_{\mathrm{hy}} \cdot HP_{4C} \tag{17}$$

$$\frac{dHP_{4C}}{dt} = k_{\mathrm{hx}} \cdot HP_{3C} - k_{\mathrm{hy}} \cdot HP_{4C} - k_{\mathrm{him}} \cdot HP_{4C} + k_{\mathrm{hk}} \cdot HP_{4n} \tag{18}$$

$$\frac{dHP_{4n}}{dt} = k_{\mathrm{him}} \cdot HP_{4C} - k_{\mathrm{hk}} \cdot HP_{4n} \tag{19}$$

$$\frac{dEH_{5C}}{dt} = k_{\mathrm{fmh1}} \cdot H5_p \cdot \left(E47_t - ME - ME_{pc} - 2 \cdot ME_{pc2} - 2 \cdot ME_{pn} - IE - EH_{5C}\right) - k_{\mathrm{bmh1}} \cdot H6_p \cdot EH_{5C} \tag{20}$$

$$\frac{dH5_{pt}}{dt} = k_{\mathrm{hsynp}} \cdot H5_{mc} - k_{\mathrm{dhes5}} \cdot H5_p - \frac{k_{\mathrm{hdyn}} \cdot H5_p \cdot H6_p}{k_{\mathrm{5mm}} + H5_p} \tag{21}$$

$$\frac{dG_{ma}}{dt} = \left(k_{\mathrm{mi}} + k_{\mathrm{bmp2}} . \mathrm{BMP2}\right) \cdot (G_{mt} - G_{ma}) \cdot ME_{pn} - k_{\mathrm{ma}} \cdot G_{ma} \cdot HP_{4n} \tag{22}$$

$$\frac{dM_m}{dt} = k_{\mathrm{msyn}} \cdot G_{ma} + J_{\mathrm{m}} \cdot (G_{mt} - G_{ma}) - k_{\mathrm{dmm}} \cdot M_m - k_{\mathrm{exmm}} \cdot M_m \tag{23}$$

$$\frac{dM_C}{dt} = k_{\mathrm{exmm}} \cdot M_m - k_{\mathrm{dmc}} \cdot M_C \tag{24}$$

$$\frac{dM_p}{dt} = k_{\mathrm{mp}} \cdot M_C - \left(k_{\mathrm{dmp}} + k_{\mathrm{dmpid}} . I_p\right). M_p - k_{\mathrm{fme}} \cdot M_p \cdot \left(E47_t - ME - ME_{pc} - 2 \cdot ME_{pc2} - 2 \cdot ME_{pn} - IE - EH_{5C}\right) + k_{\mathrm{bme}} \cdot ME \tag{25}$$

$$\frac{dME}{dt} = k_{\mathrm{fme}} \cdot M_p \cdot \left(E47_t - ME - ME_{pc} - 2 \cdot ME_{pc2} - 2 \cdot ME_{pn} - IE - EH_{5C}\right) - k_{\mathrm{bme}} \cdot ME - k_{\mathrm{fme1}} \cdot ME + k_{\mathrm{bme1}} \cdot ME_{pc} \tag{26}$$

$$\frac{dME_{pc2}}{dt} = k_{\mathrm{fme2}} \cdot ME_{pc}^2 - k_{\mathrm{bme2}} \cdot ME_{pc2} - k_{\mathrm{immep}} \cdot ME_{pc2} + k_{\mathrm{exmep}} \cdot ME_{pn} \tag{27}$$

$$\frac{dME_{pn}}{dt} = k_{\mathrm{immep}} \cdot ME_{pc2} - k_{\mathrm{exmep}} \cdot ME_{pn} \tag{28}$$

$$\frac{dM_t}{dt} = k_{\mathrm{mp}} \cdot M_C - \left(k_{\mathrm{dmp}} + k_{\mathrm{dmpid}} . I_p\right) . M_p \quad (29)$$

$$\frac{dG_{ida}}{dt} = \left(k_{\mathrm{idi}} + k_{\mathrm{bmp21}} . \mathrm{BMP2}\right) \cdot (G_{idt} - G_{ida}) - k_{\mathrm{ida}} \cdot G_{ida} \quad (30)$$

$$\frac{dI_{mn}}{dt} = k_{\mathrm{idsyn}} \cdot G_{ida} + J_{\mathrm{i}} \cdot (G_{idt} - G_{ida}) - k_{\mathrm{didmn}} \cdot I_{mn} - k_{\mathrm{exidm}} \cdot I_{mn} \quad (31)$$

$$\frac{dI_{mc}}{dt} = k_{\mathrm{exidm}} \cdot I_{mn} - k_{\mathrm{didmc}} \cdot I_{mc} \quad (32)$$

$$\frac{d\mathrm{IE}}{dt} = k_{\mathrm{fide}} \cdot I_p \cdot \left(E47_t - ME - ME_{pc} - 2 \cdot ME_{pc2} - 2 \cdot ME_{pn} - IE - EH_{5C}\right) - k_{\mathrm{bide}} \cdot IE \quad (33)$$

$$\frac{dI_t}{dt} = k_{idp} \cdot I_{mc} - k_{\mathrm{didp}} \cdot I_p \quad (34)$$

$$ME_{pc} = \left(M_t - M_p - ME - 2 \cdot ME_{pc2} - 2 \cdot ME_{pn}\right) \quad (35)$$

$$I_p = I_t - IE \quad (36)$$

$$H5_p = \left(H5_{pt} - 2 \cdot H5_{p2} - 2 \cdot HP_2 - 2 \cdot HP_{1C} - 2 \cdot HP_{2C} - 2 \cdot HP_{3C} - 2 \cdot HP_{4C} - 2 \cdot HP_{4n} - EH_{5C}\right) \quad (37)$$

Interestingly, the bifurcation diagram shown in Fig. A2b contains only the second bi-stable region in the steady state level of total Mash1 protein as a function of BMP2. Fig. A2c shows that at a pre-defined low BMP2 (BMP2=2 s.u.), the steady state level of total Mash1 remains in the high expression state and total Hes5 remains in the low Hes5 expressing state indicative of a neuronal state. Now at a pre-defined high BMP2 (BMP2=20 s.u.) dose, the steady state level of total Mash1 deterministically stays at the low expressing state and total Hes5 reaches the higher expression level representative of a gliogenic like state. Thus increase in BMP2 causes a decrease in the total Mash1 level with consequent up-regulation of total Hes5 level, which indicates acceleration of gliogenesis at high BMP2. Further, one can observe that the system can even show an oscillatory dynamics at the low Hes5 expressing state with a period of nearly 2 hr (Fig. A2c) but in the high Hes5 expressing state the oscillations are not present (Fig. A2c inset). The overall dynamics of the NSC's now is quite close to what has been observed for NSC's in CNS.

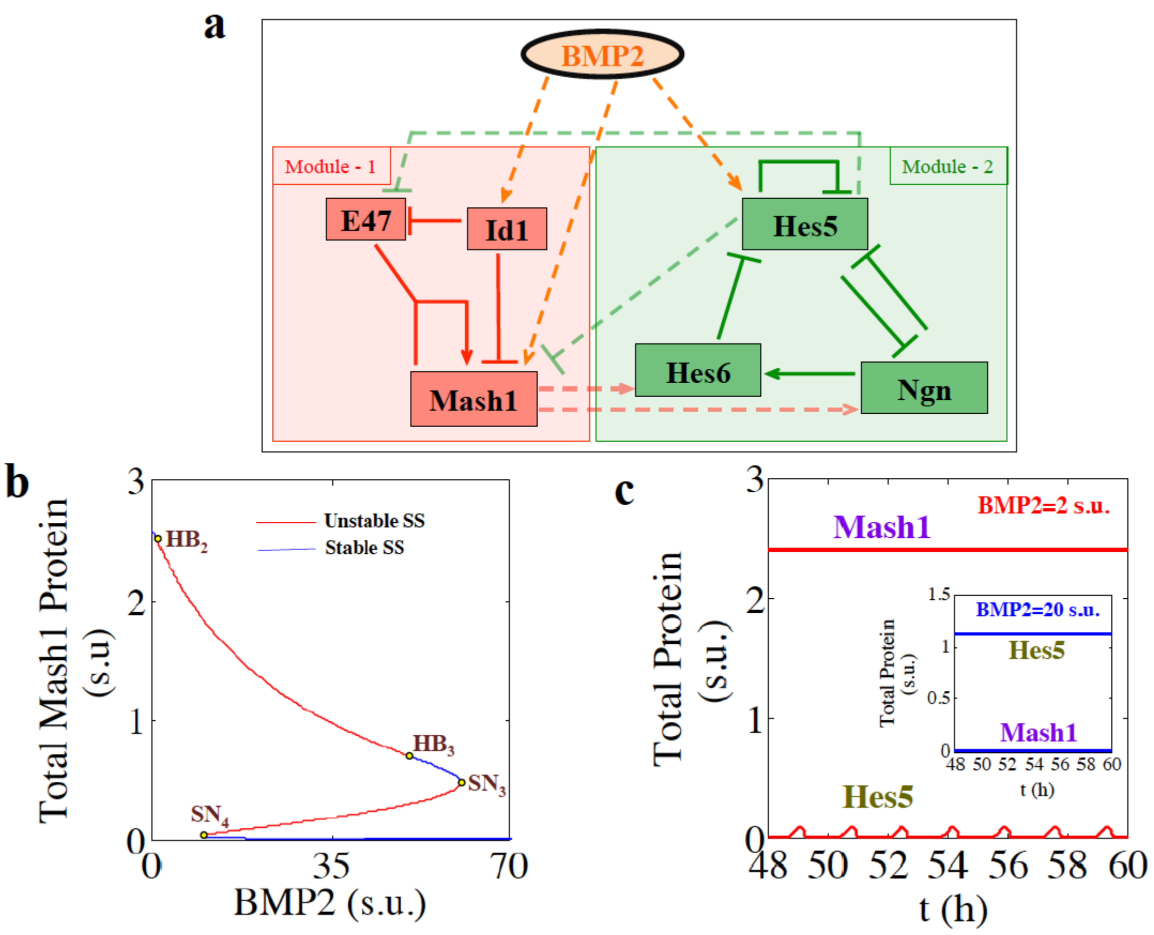

Figure A2. (a) Schematic representation of the extended model. **(b)** We plot steady state levels of total Mash1 protein as a function of BMP2 in the extended model. **(c)** Time profiles of total protein levels of Mash1 and Hes5 at BMP2=2 s.u. and BMP2=20 s.u. (inset). Parameters are given in SI Text.

The complete model after including module-3

Dynamical regulation of negative HLH factor Hes1 is illustrated in module-3 (Fig. 1, Supplementary Fig. 1). Hes1 expression is usually up regulated in response to Notch stimulation[1,20,22,26]. Once Hes1 gets dimerized it interacts with Gro/TLE that ultimately leads to Gro/TLE hyper-phosphorylation[7,8,21-24,26]. This hyper-phosphorylated complex helps Hes1 to repress (corresponds the self repression link of Hes1 in Module-3 of Fig. 1 (main text)) its own transcription in a negative feedback fashion[14,27]. Some attempts have been made beforehand to model only Hes1 auto-regulatory negative feedback by incorporating time delay in the dynamical evolution equations[13-15]. In this model, by taking the help of experimental literature, we use the appropriate molecular biology behind the negative auto-regulation of Hes1 in our model. We further incorporate the effect of hetero-dimerization of Hes1 with E47 and

cessation (corresponds the Hes1 to E47 repression link between Module-3 to Module-1 of Fig. 1 (main text)) of its activity[1,10,11,25].

In module-3 we consider *Hes1* transcription occurs in nucleus from both inactive gene (G_i) and active gene (G_a). *Hes1* mRNA ($H1_{mn}$) gets transported from nucleus to cytoplasm and translates the Hes1 protein ($H1_p$). The cytoplasmic level of *Hes1* mRNA is designated as $H1_{mc}$. The dimeric form of the Hes1 protein ($H1_{p2}$) binds with Gro/TLE protein and forms a complex (GP_2). In the complex, Gro/TLE gets phosphorylated multiple times. We consider, to keep the system simple, about four times phosphorylation of Gro/TLE in GP_2. Afterwards, the hyper-phosphorylated complex is transported to the nucleus. It is termed as P_{4n}. Hes1 represses (corresponds the self repression link of Hes1 in Module-3 of Fig. 1 (main text)) its own transcription through P_{4n}. The effect of Notch stimulation on Hes1 is incorporated in the rate constant k_{del}.

This whole module-3 is added to the model already generated by coupling module-1 and module-2 to obtain the full model (Supplementary Fig. 1) by incorporating the following additional interactions. In our model, P_{4n} inhibits *Mash1* (corresponds the Hes1 to Mash1 repression link between Module-3 to Module-1 of Fig. 1 (main text)) and *Ngn* (corresponds the Hes1 to Ngn repression link between Module-3 to Module-2 of Fig. 1 (main text)) gene activation as Hes1 known to inhibit pro-neural genes. Similarly, Ngn protein (N_{pn}) inhibits (corresponds the Ngn to Hes1 repression link between Module-2 to Module-3 of Fig. 1 (main text)) *Hes1* gene activation. $H6_p$ inhibits (corresponds the Hes6 to Hes1 repression link between Module-3 to Module-2 of Fig. 1 (main text)) the formation of GP_2. $H6_p$ also induces the proteolytic degradation (corresponds the Hes6 to Hes1 repression link between Module-3 to Module-2 of Fig. 1 (main text)) of $H1_p$. $H1_p$ forms a non-functional dimer (EH_{1C}) with E47 and $H6_p$ inhibits Hes1 to form EH_{1C}.

The governing equations, abbreviated names of different species involved in the network and parameters for the full model are depicted in SI Text and it seems to satisfactorily describe the WT as well as the mutant and over-expression phenotypes for NSC's in CNS (described in the main text). In all the gene knockout cases (Fig. 3, Supplementary Fig. 3) we use total gene=0 for the corresponding gene that is knocked

out and for the over-expression cases (Fig. 3, Supplementary Fig. 3) we over-express the corresponding total gene five times in comparison to the WT situation. Other parameters are same as provided in SI Text.

The model describes a number of experimental phenotypes for NSC's in PNS

To characterize BMP2 driven neuronal differentiation in PNS we have used the same network architecture (Supplementary Fig. 1 and SI text) with changes made in the effect of BMP2 (k_{bmp2}=100 min^{-1}, k_{bmp22}=3e-02 min^{-1}, other parameters are same as SI Text) driven Mash1 and Hes5 transcription rates. The model can reconcile the WT behavior of NSC's in PNS and can further predict various phenotypic conditions including mutants and over-expression (Supplementary Fig. 4) cases as listed in A3 Table. In all the gene knockout cases we use total gene=0 for the corresponding gene that is knocked out and for the overexpression cases we overexpress the corresponding total gene five times in comparison to the WT situation. Other parameters are same as given in SI Text (mentioned in the description of the parameters and their values and sources for CNS section).

A3 Table: Various phenotypic conditions in PNS

	Conditions	Simulated phenotype		Conditions	Simulated phenotype
1	Hes1 oscillation (2-hour cycle)	Figs. 4c-d	9	Δ *Hes5*, neurogenesis increased	Fig. S4h
2	*Hes1* mRNA oscillation (2-hour cycle)	Fig. S4a	10	Δ *Hes1*, neurogenesis increased	Fig. S4i
3	Time-delay and peak to trough ratio of *Hes1* mRNA and Hes1 protein	Fig. S4b	11	*Mash1* OE, neurogenesis increased	Fig. S4j
4	Presence of proteasome inhibitor of Hes1 protein, *Hes1* mRNA low, Hes1 protein high	Fig. S4c^{+}	12	*Ngn* OE, no significant effect	Fig. S4k
5	Δ*Mash1*, neurogenesis inhibited	Fig. S4d	13	*Hes6* OE, neurogenesis increased	Fig. S4l
6	Δ*Ngn*, complementary expressions of Mash1 and Ngn	Fig. S4e	14	*Id1* OE, neurogenesis inhibited	Fig. S4m
7	Δ *Hes6*, neurogenesis inhibited	Fig. S4f	15	*Hes5* OE, neurogenesis inhibited	Fig. S4n
8	Δ *Id1*, neurogenesis increased	Fig. S4g	16	*Hes1* OE, neurogenesis inhibited	Fig. S4o

$^{+}$ k_{dhes1}=0 min^{-1} and k_{dyn}=0 min^{-1}

The model predicts the possible routes to reconcile the developmental features of WT NSC's in PNS

Our systematic sensitivity analysis revealed that for few other pairs of parameters, we could also get a PNS like behavior (Other than shown in Fig. 4). The model predicts that for the following parameter values provided in A4 Table one can reconcile a PNS like situation as shown in Supplementary Fig. 7. Other parameters are same as SI Text (mentioned in the description of the parameters and their values and sources for CNS section). Supplementary Fig. 7a (left and middle panels) demonstrates that increase in k_{mp} (translation rate of Mash1 protein) has more effect on the saddle node SN_1 than the saddle node SN_4. The fact that k_{bmp22} and k_{mp} act in an opposite way towards SN_1 and SN_4, immediately suggests that both of them can be tuned to get the PNS like feature as shown in the Supplementary Fig. 7a (right panel). Thus, lowering the values for both k_{bmp22} and k_{mp} reinforces neurogenesis (high Mash1 expression) at high BMP2 level (BMP2=20 s.u.) and ensures gliogenesis (low Mash1 expression) at lower values (BMP2=2 s.u.) of BMP2 doses.

Supplementary Fig. 7b (left panel and middle panels) seems to suggest that both the saddle nodes SN_1 and SN_4 are more sensitive towards increase in k_{dmpid} (Id1 mediated degradation rate of Mash1 protein) in comparison to k_{bmp22}. This again indicates that by decreasing k_{bmp22} to a sufficient extent and by slightly increasing k_{dmpid}, one can enforce neuronal differentiation at high BMP2 (i.e., at BMP2=20 s.u.) and consequently onset of gliogenesis can happen at much lower (BMP2=2 s.u.) BMP2 levels (PNS like situation in Supplementary Fig. 7b (right panel)). In similar note to what we observe in Supplementary Fig. 7b, Supplementary Fig. 7c (left panel and middle panels) evidently depicts that both the saddle nodes SN_1 and SN_4 are relatively more sensitive towards increase in k_{hsynp} (translation rate of Hes5 protein) in comparison to k_{bmp22}. Thus a sufficient decrease in k_{bmp22} and a moderate increase in k_{hsynp} create a PNS like feature as shown Supplementary Fig. 7c (right panel).

A4 Table: Model predictions

Figure No.	Parameter	Value (min^{-1})
Fig. S7a	k_{bmp22}	5e-02
	k_{mp}	2.5e-02
Fig. S7b	k_{bmp22}	5e-02
	k_{dmpid}	4.5e-01
Fig. S7c	k_{bmp22}	4e-02
	k_{hsynp}	1.0

The biological relevance of decreasing the parameter k_{bmp22} has been discussed in quite elaborate fashion in the main text. Although we tried to stress the point that expression levels of different proteins and extent of the different feedback loop incorporated within k_{bmp22} are responsible for transforming the system to a PNS like dynamics, it is hard to pin point what is the exact reason behind such an observation. More experiments in this direction will be helpful to decipher this issue more clearly. On the other hand, one can easily tune the parameters k_{mp} (translation rate of Mash1 protein), k_{dmpid} (Id1 mediated degradation rate of Mash1 protein) and k_{hsynp} (translation rate of Hes5 protein) experimentally and verify these model predictions.
